# Re‐irradiation volumetric modulated arc therapy optimization based on cumulative biologically effective dose objectives

**DOI:** 10.1002/acm2.12481

**Published:** 2018-10-29

**Authors:** Nevin McVicar, Steven Thomas, Mitchell Liu, Hannah Carolan, Alanah Bergman

**Affiliations:** ^1^ Department of Medical Physics BC Cancer – Vancouver Cancer Centre Vancouver BC Canada; ^2^ Department of Radiation Oncology BC Cancer – Vancouver Cancer Centre Vancouver BC Canada

**Keywords:** adaptive replanning, linear‐quadratic model, radiobiology, VMAT dose optimization algorithms

## Abstract

The objective of this note is to introduce a clinical tool that generates ideal base plan dose distributions to enable re‐irradiation volumetric modulated arc therapy (VMAT) optimization based on cumulative biological effective dose objectives for specific organs at risk (OARs). The tool is demonstrated with a lung cancer case that required re‐irradiation at our clinic. First, previous treatment dose is deformed onto the retreatment computed tomography (CT) using commercial software. Then, the in‐house Matlab tool alters the deformed previous dose using radiobiological concepts on a voxel‐by‐voxel manner to generate an ideal base plan dose distribution. Ideal base plans that were generated using the in‐house Matlab tool were compatible with the Varian Eclipse™ treatment planning system. The tool enabled optimization of VMAT re‐irradiation plans using cumulative dose limits for OARs and all OAR cumulative dose objectives were met on the first optimization for the recurrent lung cancer case tested.

**Abbreviations**
BED: biologically effective dose
CT: computed tomography
DICOM: digital imaging and communications in medicine
DVH: dose‐volume histogram
LQM: linear‐quadratic model
OAR: organ at risk
PTV: planning target volume
SABR: stereotactic ablative radiotherapy
TPS: treatment planning system
VMAT: volumetric modulated arc therapy

## 1 INTRODUCTION

High‐dose re‐irradiation has emerged as a feasible treatment option in lung cancer patients with locoregional relapse and few other treatment options. Re‐irradiation of locoregional relapse is becoming more common as lung cancer patients continue to live longer. Modern radiotherapy inverse plan optimization and delivery technologies such as intensity modulated radiotherapy (IMRT) and volumetric modulated arc therapy (VMAT) improve sparing of organs at risk (OARs) and make high‐dose re‐irradiation more feasible. Still, re‐irradiation introduces several complexities and plan optimization is cumbersome. For example, patient anatomy changes and dose accumulation to OARs must be accounted for. The challenge of changing patient anatomy has largely been met through rigid and deformable image registration (IR) algorithms that enable IR‐guided dose transfer between planning computed tomography (CT) scans.^1^ In addition, computer software capable of converting voxel doses using the linear‐quadratic model (LQM)^2^ to calculate biologically effective dose (BED) distributions is now commercially available.^3^, ^4^ Converting previously delivered physical dose to BED allows radiation oncologists to account for nonlinear biological response to differing dose‐per‐fraction.^4^ Moreover, BED distributions are theoretically additive according to the LQM — meaning distributions from separate courses of treatment can be summed to quantify cumulative BED for each voxel.^3^, ^4^, ^5^, ^6^ The mechanistic LQM is often considered to be over simplistic^7^; however, it is almost universally used to adjust for fraction size because it is practical, biologically based and acceptably accurate for dose‐per‐fractions below 15 Gy.^2^, ^8^

Based on recent reports, a growing number of radiation oncologists are using cumulative BED distributions to guide their re‐irradiation plan evaluation.^3^, ^6^ Several institutions have reported OAR toxicity along with cumulative BED metrics in lung cancer patients receiving re‐irradiation,^9^, ^10^ with some instances of severe toxicities after delivering high cumulative dose to the aorta,^11^, ^12^ esophagus,^9^, ^13^ and lungs.^14^, ^15^, ^16^ This clinical evidence has motivated radiation oncologists to prescribe cumulative BED limits for specific OARs.^3^, ^6^ While IR and dose accumulation techniques improve plan evaluation and clinical decision‐making, re‐irradiation plan optimization remains difficult because commercial treatment planning systems (TPSs) are not designed to optimize based on cumulative BED objectives as outlined below.

Throughout VMAT optimization, TPSs display dose volume histograms (DVHs) for OARs and planning target volumes (PTVs). Visualization of evolving DVHs during optimization enables dosimetrists to influence the optimization by interactively adjusting dose objectives. The ability to monitor and adjust dose objectives throughout optimization is essential to the quality and efficiency of VMAT planning.^17^ For initial irradiation plans, the evolving DVHs display total physical dose delivered over all planned fractions. For re‐irradiation optimization, TPSs allow for VMAT optimization using previously delivered dose as a base plan. Throughout optimization with a base plan, displayed DVHs result from the sum of base plan and optimizing plan dose distributions and are henceforth referred to as sum DVHs. Re‐irradiation plan optimization using previously delivered physical dose as a base plan may be useful when initial and re‐irradiation plans deliver the same dose‐per‐fraction. However, when significantly different dose‐per‐fractions are used, sum DVHs become nonsense since nonlinear radiobiological effects are not accounted for. Thus, plan optimization is cumbersome because dosimetrists lose the ability to adjust objectives based on evolving DVHs. Furthermore, after each optimization cycle, dosimetrists or physicists must convert dose to BED to determine whether cumulative BED objectives are satisfied.^3^, ^6^ Application of base plan dose is further limited in re‐irradiation scenarios because optimization of uniform target dose is impossible due to the presence of heterogenous base plan dose within the new PTV, which is henceforth referred to as PTV_2_.

In this technical note, we introduce a clinical tool that enables direct VMAT optimization of cumulative dose objectives using an ideal base plan dose that is compatible with a commercial TPS. Our proposed tool manipulates previously delivered dose to circumvent conventional base plan limitations and help guide VMAT optimization of re‐irradiation. Specifically, ideal base plans restore the ability to monitor whether cumulative dose objectives are achieved using the evolving sum DVHs throughout optimization. The tool is demonstrated retrospectively for a lung cancer case where re‐irradiation was prescribed.

## 2 METHODS AND RESULTS

### 2.A Patient data

A patient with recurrent lung cancer previously treated and retreated at our clinic was used in this study. An initial course of VMAT was used to deliver a prescription of 60 Gy in 30 fractions to a primary lung PTV (PTV_1_). Approximately 3 yr later, a second primary lesion was discovered and a course of stereotactic ablative radiotherapy (SABR) was prescribed to deliver 48 Gy in four fractions to the PTV_2_. The clinically delivered initial course dose, referred to as *D*_1_, was used in this study along with the initial and retreatment CTs, referred to as CT_1_ and CT_2_, respectively. To guide the optimization of the re‐irradiation dose distribution (*D*_2_), a radiation oncologist retrospectively provided a list of OAR‐specific α/β ratios and prioritized cumulative dose limits which are listed in both physical dose (Gy per 4 fractions) and BED (Gy_α/β_) in Table 1.^18^

**Table 1:** Summary of biological effective dose to organs at risk

Priority	Organ	α/β	BED metric	BED_1_	BED_1_onCT2_	BED_2_	BED sum	Cumulative dose limits	
								BED (Gy_α/β_)	Physical (Gy/4 fx)
1	Spinal canal	2	*D*_max_ (Gy_2_)	47.2	46.9	12.1	47.1	*D*_max_ < 110.5	*D*_max_ < 26
2	PBT^a^	3	*D*_max_ (Gy_3_)	21.8	21.5	32.2	34.7	*D*_max_ < 135.7	*D*_max_ < 34.8
3	Esophagus	3	*D*_max_ (Gy_3_)	100.5	104.3	12.7	104.4	*D*_max_ < 105	*D*_max_ < 30
4	Lungs‐PTV^b^	3	*V*_53.3Gy3_ (%)	3.5	1.8	1.3	3.3	*V*_53.3_ < 10%	V_20_ < 10%
5	Heart	3	*D*_max_ (Gy_3_)	104.6	107.8	40.5	107.9	*D*_max_ < 130.3	*D*_max_ < 34
6	Skin	3	*D*_max_ (Gy_3_)	47.3	48.5	99.5	105.0	*D*_max_ < 144	*D*_max_ < 36

BED_1_ doses are reported using CT_1_ contours.

BED_1_onCT2_ doses are reported using CT_2_ contours after warping BED_1_ onto CT_2_.

BED_2_ and BED sum doses are reported using CT_2_ contours.

a PBT, proximal bronchial tree.

b Lungs‐PTV contours are different in CT_1_ and CT_2_ due to different PTVs.

### 2.B CT_1_‐to‐CT_2_ deformable image registration

**Figure 1:**
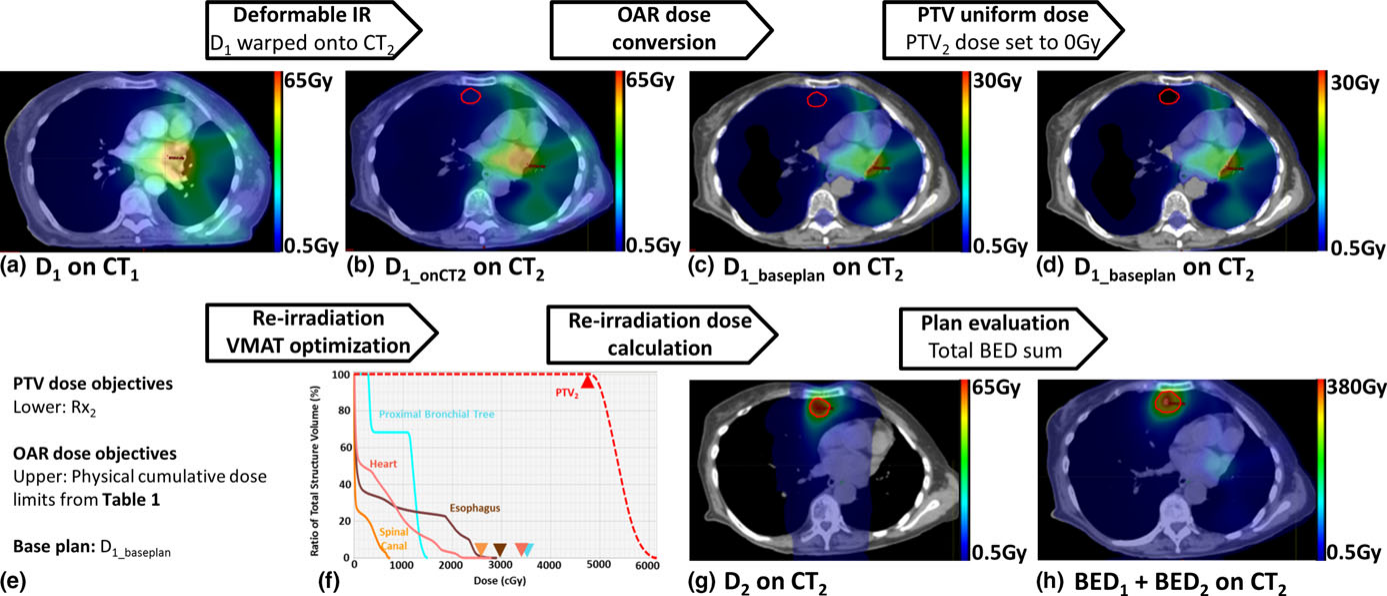
Workflow for retreatment VMAT planning and evaluation: (a) Initial treatment dose (*D*_1_) calculated on initial treatment planning CT_1_. (b) *D*_1_ warped onto re‐irradiation CT_2_ to produce *D*_1_onCT2_. (c) Dose within OARs is altered using radiobiological concepts to generate an ideal base plan. (d) Dose uniformity within retreat PTV_2_ is achieved by setting dose to 0 Gy to produce *D*_1_baseplan_. (e, f) Re‐irradiation VMAT plan is optimized using cumulative OAR and PTV dose objectives that account for base plan, *D*_1_baseplan_. During optimization, sum DVHs accurately reflect whether dose objectives are achieved. (g) PTV_2_ and OAR dose assessment using retreatment dose (*D*_2_). (h) Finally, *D*_1_onCT2_ and *D*_2_ are converted to BED and summed to assess cumulative BED to OARs. In (b–d) and (g–h), PTV_2_ is outlined in red.

An overview of the re‐irradiation planning workflow that incorporates the proposed tool is shown in Fig. 1. First, CT_1_‐to‐CT_2_ deformable IR was performed and *D*_1_ was warped onto CT_2_ to generate *D*_1_onCT2_ using a commercial intensity‐based IR algorithm (MIM Software Inc., Cleveland, OH, USA). As per clinical protocol, deformable IR was performed by a medical physicist and verified independently by a second medical physicist and a radiation oncologist. Deformable IR errors are not explicitly quantified or accounted for in this workflow.

### 2.C Generation of base plan

The tool is an in‐house Matlab (The Mathworks Inc., Natick, MA, USA) program that manipulates *D*_1_onCT2_ to generate an ideal base plan dose for re‐irradiation optimization, referred to as *D*_1_baseplan_. The tool requires user‐specified initial course prescription (*R*_x1_) in Gy, the number of fractions for initial (*n*_1_) and re‐irradiation (*n*_2_) courses, and OAR‐specific priorities and α/β ratios. In addition, the tool requires a cumulative dose limit (*D*_L_) specified in Gy per *n*_2_ fractions (*D*_L_ = *d*_L_ × *n*_2_) for each OAR. The tool allows for one *D*_L_ per OAR and *D*_L_ must represent a maximum point dose limit (e.g_.,_ *D*_max_ < *D*_L_) or a maximum dose to a specified volume limit (e.g., *V*_DL_ ≤ % OAR volume).

The tool requires the digital imaging and communications in medicine (DICOM) structure file associated with CT_2_ to label each voxel in *D*_1_onCT2_ as a specific OAR or PTV_2_. In cases of overlapping contours, voxels are labeled as the highest priority structure. Since TPSs display the sum DVH (*D*_1_baseplan_ + optimizing *D*_2_) throughout optimization in Gy per *n*_2_ fractions, *D*_1_baseplan_ voxel dose is also specified in Gy per *n*_2_ fractions.

#### 2.C.1 OAR base plan dose

For each OAR, *D*_1_onCT2_ is converted voxel‐by‐voxel to a physical dose in Gy per *n*_2_ fractions equal to the *radiobiological fraction* of *D*_L_ previously delivered by initial treatment. To do this, *D*_L_ is converted to BED (BED_L_) using the LQM formalism^2^, ^19^:

$${\text{BED}}_{L}=d_{L}\times n_{2}\left(1+\frac{d_{L}}{\alpha /\beta }\right).$$

Similarly, *D*_1_onCT2_ is converted to BED (BED_1_) for all voxels. Then, for each voxel with BED_1_ < BED_L_, the *allowed* re‐irradiation voxel dose is defined as *D*_allowed_ = *d*_allowed_ × *n*_2_, with *d*_allowed_ calculated using the equality:

$${\text{BED}}_{L}-{\text{BED}}_{1}\equiv {\text{BED}}_{\mathrm{allowed}}=d_{\mathrm{allowed}}\times n_{2}\left(1+d_{\frac{\mathrm{allowed}}{\alpha /\beta }}\right),$$

and then, *D*_1_baseplan_ voxel dose is set to *D*_L_ − *D*_allowed_.

Here, we highlight two important points regarding voxels with BED_1_ < BED_L_ that receive exactly *D*_allowed_ during re‐irradiation: (a) *D*_1_baseplan_ + *D*_allowed_ = *D*_L_ and therefore, these voxels are easily monitored on the sum DVH throughout optimization and (b) BED_1_ + BED_allowed_ = BED_L_; hence, the sum DVH value at *D*_L_ accurately portrays whether *D*_L_ is satisfied or not. Furthermore, voxels with BED_1_ < BED_L_ that receive under *D*_allowed_ will appropriately appear below *D*_L_ on the sum DVH throughout optimization, whereas voxels with BED_1_ < BED_L_ that receive over *D*_allowed_ appear above *D*_L_. Hence, the advantage to the dosimetrist is that evolving sum DVHs reliably illustrate whether cumulative dose objectives are met throughout optimization.

For OARs where *D*_L_ represents a maximum dose to a specified volume, some voxels will have BED_1_ ≥ BED_L_. For these voxels, *D*_1_baseplan_ voxel dose is set to the isoeffective dose delivered in *n*_2_ fractions, referred to as *D*_1_n2_ where *D*_1_n2_ = *d*_1_n2_ × *n*_2_, with *d*_1_n2_ calculated using the equality:

$$\begin{array}{cc} {\text{BED}}_{1}= & d_{1}\times n_{1}\left(\frac{1+d_{1}}{\alpha /\beta }\right)\\ = & d_{1\_n2}\times n_{2}\left(1+\frac{d_{1\_n2}}{\alpha /\beta }\right) \end{array}$$

The isoeffective dose conversion essentially scales *D*_1_onCT2_ dose to the re‐irradiation fractionation such that it will correctly appear above *D*_L_ on the sum DVH throughout the entire optimization. It must be recognized that none of the OAR sum DVH values represent true physical or biological dose when *D*_1_baseplan_ is used. However, each sum DVH may be regarded as a LQM‐scaled approximation that accurately reports whether *D*_L_ is satisfied.

#### 2.C.2 PTV_2_ base plan dose

All voxels labeled as PTV_2_ are set to an arbitrary uniform dose in *D*_1_baseplan_. For practical purposes, PTV_2_ voxel doses are set to 0 Gy in this case. The uniform base plan dose in PTV_2_ is required to enable optimization of a uniform re‐irradiation dose to PTV_2_.

Finally, *D*_1_baseplan_ is saved as a DICOM dose file, using the original dose files’ DICOM header information. The Matlab‐generated DICOM base plan dose files are then imported to the version 13.0 Eclipse^™^ TPS.

### 2.D Re‐irradiation VMAT optimization

The re‐irradiation plan consisted of two full coplanar arcs placed at the center of PTV_2_. For the recurrent lung case, *D*_1_baseplan_ was generated using tool inputs: *R*_x1_/*n*_1_ = 60/30, *n*_2_ = 4 along with all OAR‐specific priorities, α/β, and *D*_L_ listed in Table 1. Optimization dose objectives were set according to prioritized cumulative dose limits in Gy per *n*_2_ fractions listed in Table 1. Since PTV_2_ voxel dose is 0 Gy in *D*_1_baseplan_, PTV_2_ lower dose objective is set to *R*_x2_ (48 Gy).

### 2.E. Re‐irradiation plan evaluation

After optimization, *D*_2_ and *D*_1_onCT2_ were converted to BED using MIM software and the cumulative BED was calculated. Cumulative dose limits were satisfied and OAR‐specific sum BED metrics are reported in Table 1. For the re‐irradiation plan only, the percent volume of PTV_2_ that received ≥100% of the *R*_x2_ (V_100%_) was 99.9% and the *D*_max_ was 128% (61.6 Gy), which is acceptable for SABR treatment at our clinic.

## 3 DISCUSSION

To our knowledge, this is the first strategy to use the previously delivered dose and radiobiological concepts to generate an ideal three‐dimensional base plan for re‐irradiation optimization. The impact of the ideal base plan on the final optimized dose distribution is outlined further here. In serial OARs where *D*_L_ represents a maximum point dose limit, voxels that previously received higher doses will be preferentially spared compared to voxels that received lower doses in order to achieve cumulative *D*_max_ < *D*_L_ in each voxel. This effect is in agreement with other published re‐irradiation studies that limited cumulative maximum point dose for serial OARs such as aorta, esophagus, and spinal cord. Conversely, in parallel OARs where *D*_L_ represents a maximum dose to a specified volume limit, voxels that previously received doses > *D*_L_ will be preferentially irradiated compared to voxels that received doses < *D*_L_ in order to minimize cumulative *V*_DL_. For example, when the Lung‐PTV dose objective is *V*_20Gy_ ≤ 10%, voxels in the lung that previously received >20 Gy are not spared during re‐irradiation optimization. Again, this effect is in agreement with previous findings that patients retreated with infield relapses experienced lower rates of pneumonitis compared with those retreated with out of field relapses,^14^, ^16^ which suggests that previously irradiated lung tissue may have fibrosis and be less susceptible to radiation pneumonitis.^20^ Hence, our tool enables serial and parallel OARs to be optimized in different manners based on the type of dose objective, *D*_L_, used. Key limitations of our algorithm are that it only supports one objective per OAR and it cannot support cumulative mean dose objectives.

As standard fractionation schemes (1.8–2 Gy per fraction) are replaced with hypofractionated stereotactic ablative radiotherapy (>6 Gy per fraction), oncologists increasingly rely on the LQM to derive optimal re‐irradiation fractionation schemes and OAR dose objectives. The use of LQM‐derived cumulative BED dose constraints is controversial because the additivity of BED distributions is theoretical and very difficult to validate clinically. In this work, the inherent uncertainty of LQM‐derived BED values is not explicitly quantified and cumulative BED distributions should only serve to compliment careful judgment of the treating physician. Moreover, the basic LQM formalism used in this technical note does not explicitly account for the impact of ablation and/or regeneration of surrounding vasculature and stromal tissue, which is especially relevant in re‐irradiation scenarios. It may be appropriate to adapt the proposed tool to employ more complex models (e.g., to account for tissue recovery between treatment courses) if it is deemed necessary by the treating physician.^7^, ^21^ Finally, planned re‐irradiation dose distributions must always be evaluated alone in addition to the cumulative BED distribution prior to re‐irradiation commencement.

In this work, a tool was shown to facilitate optimization of VMAT re‐irradiation plans using cumulative dose limits for OARs. Ideal base plans generated using our tool were compatible with the Varian Eclipse^™^ TPS and similar tools could be developed for other TPSs that accommodate base plans. The presented tool may also be used for inverse‐planning of IMRT plans as long as base plans can be used during optimization. Although the underlying algorithm used to create the ideal base plan is complex, the tool is clinically practical because it only requires simple OAR‐specific inputs to generate an ideal base plan for each patient. Furthermore, use of the ideal base plan eliminates time‐consuming iterations of plan optimization, dose conversion to BED and BED accumulation to verify whether cumulative dose objectives are achieved or not. Future work will aim to incorporate representation of dosimetric uncertainties associated with deformable IR errors during cumulative dose evaluation.^22^ Furthermore, a script will be developed using Eclipse scripting Application Programming Interface to streamline clinical use of this tool. Finally, we will investigate potential improvements in planning time required and overall plan quality introduced by the proposed planning approach.

## CONFLICT OF INTEREST

The authors declare no conflict of interest.
